# Editorial: The role of teachers' emotions in students' outcomes: From the perspective of interpersonal emotions

**DOI:** 10.3389/fpsyg.2022.1075110

**Published:** 2022-12-02

**Authors:** Zhongling Pi, Harrison Hao Yang, Wenli Chen, Xiangen Hu, Xiying Li

**Affiliations:** ^1^Key Laboratory of Modern Teaching Technology, Ministry of Education, Shaanxi Normal University, Xi'an, China; ^2^School of Education, State University of New York, Oswego, NY, United States; ^3^National Institute of Education, Nanyang Technological University, Singapore, Singapore; ^4^Department of Psychology, University of Memphis, Memphis, TN, United States

**Keywords:** teachers, emotions, interpersonal emotions, students' outcomes, emotion labor

## Teachers' emotions

Emotions are part of our daily life. Emotions are feelings from judgments relative to specific events and can be intense and directed (Linnenbrink-Garcia et al., 2016). Being a teacher is frequently described as an emotional profession (Frenzel et al., 2016, 2018; Sutton, 2004). Teachers experience various discrete emotions in response to different situations, including interactions with others (e.g., students, colleagues, principals, and parents), reactions to teaching events, and appraisals of teaching events and the educational system (Sha et al.; Shen et al.). Teachers produce either positive emotions (e.g., pride, enjoyment, satisfaction, and happiness) or negative emotions (e.g., boredom, anger, anxiety, and frustration) or mixed emotions in each of these situations. Therefore, it is not surprising that teachers report experiencing various discrete emotions.

However, not all these emotions are always appropriate in the teaching context, as each discrete emotion is accompanied by unique actions and feelings (Cheng et al.). Teacher emotions are multi-faceted processes involving cognition, emotional experience, emotional arousal, and emotional behaviors and actions within the teaching context (Frenzel et al., 2016). For example, teachers who feel pride in their students may be confident, arousal, alert, and have positive action tendencies in their jobs. By contrast, teachers who feel boredom may produce spiritless facial expressions and postures and even experience job burnout. To be professional, teachers try their best to present an appropriate emotional image to their students by regulating their emotions (Sha et al.; Shen et al.).

Teachers' emotions can be classified by their valence (e.g., positive, negative) and physiological arousal (e.g., activating, deactivating; Cheng et al.). It is commonly believed that teachers should present positive emotions (Cheng et al.; Wang et al.). Specifically, teachers' positive emotions elicit a positive classroom climate and good relationships with others, resulting in good educational outcomes (e.g., teachers' occupational wellbeing and students' motivation and learning performance).

Teachers' emotions provide essential information about their feelings, intentions, or motives, thus enabling the students to respond adequately and adapt their behaviors (Pekrun, 2006; Jennings and Greenberg, 2009; Reyes et al., 2012; Keller and Becker, 2021). For example, when students gratuitously shut down during class, it is better for teachers to present happy or angry emotion. If teachers present happy, it is difficult for students to recognize the negative consequences of gratuitously shutting down during the class. The study by Cheng et al. compared the effects of teachers' positive, negative, and neutral emotions. They found that the teacher's positive emotions enhanced students' self-reported pleasure, the teacher's negative emotions enhanced students' productivity, and the teacher's neutral emotions enhanced students' collaborative satisfaction and a greater willingness to continue collaborating with their group. Therefore, researchers should further test whether teachers' positive emotions inevitably lead to good educational outcomes and whether their negative emotions are not.

## The influences of teachers' emotions

Teachers' emotions influence not only their occupational health and wellbeing but also students' learning and development (Liu and Wang; Sha et al.; Valentín et al.). The objective of the Research Topic entitled “*The Role of Teachers' Emotions in Students' Outcomes: From the Perspective of Interpersonal Emotions*” is to systematically explore the effects of teachers' emotions on students' outcomes. It aimed to understand the antecedent variables, consequence variables, and the mechanism regarding teachers' emotions in various settings (e.g., traditional face-to-face classroom and video lectures) from a dynamic perspective.

Regarding the influences of teachers' emotions on their outcomes, the studies by Sha et al. and Shen et al. found that teachers' emotional intelligence influenced their wellbeing and mental health, which was mediated by cognitive reappraisal, expression repression, and perceived organizational justice. Regarding the influences of teachers' emotions on students' outcomes, the study by Liu and Wang tested the effect of teachers' emotions on preschool children's social behaviors.

Some studies in this Research Topic also recognize the importance of teachers' emotions in online learning. For example, the study by Wu et al. observed that one of the most significant differences between different presentation styles was the number of emotional words used by instructors in MOOCs. Furthermore, the studies by Valentín et al. and Wang explored whether teachers' enhanced emotions influenced students' learning from texts and video lectures. They found that teachers' enhanced emotions reduced students' cognitive load and improved their motivation and learning performance.

The research studies in this Research Topic make significant contributions to the area of teachers' emotions. Furthermore, these studies also have both theoretical and practical implications. It is suggested that teachers' emotions cannot be regarded as isolated from social, cultural, and political environments, but they are intertwined, a process called emotional transmission in the teaching context (Frenzel et al., 2018). Therefore, teachers' emotions are dynamic rather than static. However, most previous studies regarded teachers' emotions as a static variable by measuring their emotions at one time and testing their relationships with other variables (e.g., students' emotional responses; Wang et al.). A transmissive and dynamic perspective on the possible roles of teachers' emotions is still lacking. Further work should understand the role of teachers' emotions in educational contexts by dynamic measures (e.g., experience sampling).

## Author contributions

All authors listed have made a substantial, direct, and intellectual contribution to the work and approved it for publication.

## Funding

This work was supported by the Fundamental Research Funds for the Central Universities (2022ZYYB22).

## Conflict of interest

The authors declare that the research was conducted in the absence of any commercial or financial relationships that could be construed as a potential conflict of interest.

## Publisher's note

All claims expressed in this article are solely those of the authors and do not necessarily represent those of their affiliated organizations, or those of the publisher, the editors and the reviewers. Any product that may be evaluated in this article, or claim that may be made by its manufacturer, is not guaranteed or endorsed by the publisher.

## References

[B1] Frenzel A. C. Becker-Kurz B. Pekrun R. Goetz T. Lüdtke O. (2018). Emotion transmission in the classroom revisited: A reciprocal effects model of teacher and student enjoyment. J. Educ. Psychol. 110, 628–639. 10.1037/edu0000228

[B2] Frenzel A. C. Pekrun R. Goetz T. Daniels L. M. Durksen T. L. Becker-Kurz B.. (2016). Measuring enjoyment, anger, and anxiety during teaching: The Teacher Emotion Scales (TES). Cont. Educ. Psychol. 46, 148–163. 10.1016/j.cedpsych.2016.05.003

[B3] Jennings P. A. Greenberg M. T. (2009). The prosocial classroom: Teacher social and emotional competence in relation to student and classroom outcomes. Rev. Educ. Res. 79, 491–525. 10.3102/0034654308325693

[B4] Keller M. M. Becker E. S. (2021). Teachers' emotions and emotional authenticity: do they matter to students' emotional responses in the classroom? Teach. Teach. 27, 404–422. 10.1080/13540602.2020.1834380

[B5] Linnenbrink-Garcia L. Patall E. A. Pekrun R. (2016). Adaptive motivation and emotion in education. Pol. Ins. Behav. Brain. Sci. 3, 228–236. 10.1177/2372732216644450

[B6] Pekrun R. (2006). The control-value theory of achievement emotions: Assumptions, corollaries, and implications for educational research and practice. Educ.Psychol. Rev. 18, 315–341. 10.1007/s10648-006-9029-922364457

[B7] Reyes M. R. Brackett M. A. Rivers S. E. White M. Salovey P. (2012). Classroom emotional climate, student engagement, and academic achievement. J. Educ. Psychol. 104, 700–712. 10.1037/a002726821400208

[B8] Sutton R. E. (2004). Emotional regulation goals and strategies of teachers. Soc. Psychol. Educ. 7, 379–398. 10.1007/s11218-004-4229-y

